# Can Volatile Organic Metabolites Be Used to Simultaneously Assess Microbial and Mite Contamination Level in Cereal Grains and Coffee Beans?

**DOI:** 10.1371/journal.pone.0059338

**Published:** 2013-04-16

**Authors:** Ângelo C. Salvador, Inês Baptista, António S. Barros, Newton C. M. Gomes, Ângela Cunha, Adelaide Almeida, Silvia M. Rocha

**Affiliations:** 1 Departament of Chemistry, QOPNA, University of Aveiro, Campus Universitário de Santiago, Aveiro, Portugal; 2 Departament of Biology and CESAM, University of Aveiro, Campus Universitário de Santiago, Aveiro, Portugal; Dowling College, United States of America

## Abstract

A novel approach based on headspace solid-phase microextraction (HS-SPME) combined with comprehensive two-dimensional gas chromatography–time-of-flight mass spectrometry (GC×GC–ToFMS) was developed for the simultaneous screening of microbial and mite contamination level in cereals and coffee beans. The proposed approach emerges as a powerful tool for the rapid assessment of the microbial contamination level (*ca*. 70 min *versus ca*. 72 to 120 h for bacteria and fungi, respectively, using conventional plate counts), and mite contamination (*ca*. 70 min *versus ca*. 24 h). A full-factorial design was performed for optimization of the SPME experimental parameters. The methodology was applied to three types of rice (rough, brown, and white rice), oat, wheat, and green and roasted coffee beans. Simultaneously, microbiological analysis of the samples (total aerobic microorganisms, moulds, and yeasts) was performed by conventional plate counts. A set of 54 volatile markers was selected among all the compounds detected by GC×GC–ToFMS. Principal Component Analysis (PCA) was applied in order to establish a relationship between potential volatile markers and the level of microbial contamination. Methylbenzene, 3-octanone, 2-nonanone, 2-methyl-3-pentanol, 1-octen-3-ol, and 2-hexanone were associated to samples with higher microbial contamination level, especially in rough rice. Moreover, oat exhibited a high GC peak area of 2-hydroxy-6-methylbenzaldehyde, a sexual and alarm pheromone for adult mites, which in the other matrices appeared as a trace component. The number of mites detected in oat grains was correlated to the GC peak area of the pheromone. The HS-SPME/GC×GC–ToFMS methodology can be regarded as the basis for the development of a rapid and versatile method that can be applied in industry to the simultaneous assessment the level of microbiological contamination and for detection of mites in cereals grains and coffee beans.

## Introduction

The food industry suffers enormous financial losses due to complains arising out of off-flavours, which also leads to the loss of consumers and suppliers confidence [1]. Furthermore, the globalization of the markets implies deep management and control of the process as many kinds of foods are processed and consumed far away from the site where they are cultivated. Apart from off-flavours, serious health problems can arise, due to the biological deterioration as a possible consequence of storage of those grains. While in storage, this biological deterioration can result of several pests such as insects, rodents, mites and microorganisms (especially fungi).

Microbial volatile metabolites produced during storage and/or processing of cereals or coffee have been used as markers of microbial contamination [2]–[4]. The metabolite volatile profile seems to be closely related to the product safety and quality. For example, the fungal metabolites can be related with the fungal specie and the type of food matrix contaminated [5]. An extensive range of common volatiles linked to microbial spoilage was reported, comprising a large diversity of alcohols, ketones, aldehydes, esters, carboxylics acids, lactones, terpenes, sulphur and nitrogen compounds [6]. The most common volatiles associated to microbial contamination in cereals and other types of foods are 2-methyl-1-propanol, 3-methyl-1-butanol, 1-octanol, 1-octen-3-ol, 2-butanone, 3-octanone, 2-hexanone, 2-heptanone, 2-methylisoborneol, geosmin, limonene, dimethyl disulfide and 3-methylfuran [2], [6]–[12]. The information on the volatile profiles of other organism associated to food spoilage such as microscopic invertebrates is extremely scarce, although mites are known to produce volatile compounds associated to specific “minty” odours in contaminated foods [13].

Rapid and reproducible approaches for screening the volatile biological metabolites in foods are an emerging concern, since the conventional microscope observations and culture-based methods, both used to detect microorganisms and the first to detect mites, are time consuming, and laborious [14]. A wide range of methods have been used to extract volatile and semi-volatile compounds, some of them based on solvent extraction, but since the 1990's, solid phase microextraction (SPME) has been extensively used. SPME is a rapid, easy, solvent-free and sensitive extraction/concentration technique [15]. For the analysis, one-dimensional gas chromatographic (1D–GC) processes are widely applied in food products, although long GC runs are needed to achieve high separation power and this technique typically shows peaks that are the result of two or more co-eluted compounds. Considerable research has been dedicated to improve the resolving power of a GC system, where one possibility is to couple, through an interface (modulator), two independent columns, *i.e.*, with different stationary phases, named as comprehensive two-dimensional gas chromatography (GC×GC) [16], [17]. The separation in first dimension (^1^D) is usually driven by the boiling point properties and polarity in the second one (^2^D). This technique shows a great potential, as it grants high degree of separation, becoming a suitable solution for the analysis of target compounds in complex matrices. GC×GC–ToFMS has been successfully used in several fields of analysis, including food matrix [18]–[20]. Moreover, the use of SPME combined with GC×GC–ToFMS for the screening of food microbial contaminants was already been used, as in moisture damage in cacao beans and for the evaluation of cucumber spoilage, revealing a higher sensitivity [21], [22].

This work reports the development of an approach based on headspace (HS)-SPME combined with GC×GC–ToFMS for the screening of biological (microbial and mites) contamination level in solid foods. A full-factorial design for the optimization of SPME experimental parameters was developed using five standards reported as microbial growth markers [8]. The developed methodology was then applied to real matrices: grains of three types of rice (rough, brown, and white rice), oat and wheat and also to green and roasted coffee beans. Simultaneously, the evaluation of microbial and mites contamination was performed by quantification of colonies (total aerobic microorganisms, yeasts and moulds) and by optical microscopy counts, respectively.

## Materials and Methods

### Samples

Seven types of samples were analysed: green and roasted coffee beans (*Coffea arabica*), rough rice (unprocessed raw rice), brown rice (unpolished rice), and white rice (*Oryza sativa* L.), unprocessed raw oat (*Avena sativa*) and unprocessed raw wheat (*Triticum aestivum*). The samples were supplied from local warehouses who have kept them in silos until the commercialization and/or transformation. After sampling they were stored in the dark, under cool and dry conditions, until analysis.

### Reagents and Standards

For the experiments hereby reported, five chemical standards were used: 3-octanone (99%; Aldrich-Chemie; Steinheim, Germany), 1-octanol (96%; Merck-Schuchardt; Darmstadt, Germany), 1-octen-3-ol (98%; Aldrich Chemical; Milwaukee, U.S.A), 3-methyl-1-butanol (≥99%; Aldrich-Chemie; Steinheim, Germany), geosmin (98%; Wako; Neuss, Germany). A stock solution was prepared with 3-octanone (120 mg L^−1^), 1-octanol (59 mg L^−1^), 1-octen-3-ol (93 mg L^−1^), 3-methyl-1-butanol (180 mg L^−1^), and geosmin (1.8 mg L^−1^) in absolute ethanol, and stored in a glass flask at 4°C.

### Full-factorial Design for Optimization of SPME Parameters

Three SPME experimental parameters were tested: temperature and time of extraction and SPME coating fibre. The SPME holder for manual sampling and fibres were purchased from Supelco (Aldrich, Bellefonte, PA, USA). Four coating fibres were used: 85 µm polyacrylate coating (PA), 100 µm polydimethylsiloxane coating (PDMS), 65 µm polydimethylsiloxane/divinylbenzene coating (PDMS/DVB) and 50/30 µm divinylbenzene/carboxen/polydimethylsiloxane coating (DVB/CAR/PDMS). SPME fibres were preconditioned in the GC injector, according to the recommendation of the manufacturer and daily conditioned for 10 min at 250°C.

An aliquot of 100 µL of the stock solution containing the five chemical standards was placed in a 120 mL glass vial, and the vial was capped with a PTFE septum and an aluminium cap (Chromacol Ltd., Herts, UK). After the closure of the sample vials, the SPME fibre was manually inserted into the sample vial headspace for 10 and 30 min at 30.0, 40.0 and 50.0°C (±0.1°C) in a water bath. This procedure was repeated in triplicate for each condition tested. All combinations of extraction time and temperature were tested with the four coating fibres. The analyses were carried out by gas chromatography–quadrupole mass spectrometry (GC–qMS). Blanks, corresponding to the analysis of the coating fibre not submitted to any extraction procedure, were run between sets of three analyses.

### Analysis of Rice, Wheat and Oat Grains and Coffee Beans

For HS-SPME assay, aliquots of 6.5–16 g of each sample, corresponding to a volume *ca*. 20 mL (1/β ratio of 0.5) were placed into a 60 mL glass vial, and the vial was capped with a PTFE septum and an aluminium cap. The vial was placed in a thermostated water bath at 50.0°C, and then the DVB/CAR/PDMS fibre was inserted in the headspace during the 30 min of extraction. Three independent assays were conducted for each type of grain. The analyses were carried out by GC×GC–ToFMS. Blanks, corresponding to the analysis of the coating fibre not submitted to any extraction procedure, were run between the sets of three analyses.

### GC-qMS Analysis

After the extraction/concentration step of the five standards under study from the stock solution, the SPME coating fibre was manually introduced into the GC injection port at 250°C where it was maintained for 3 min for desorption. The injection port was lined with a 0.75 mm I.D. splitless glass liner. The analysis of volatiles extracted by HS-SPME was carried in an Agilent Technologies 6890 N Network gas chromatograph, equipped with a 60 m×0.25 mm I.D., 0.25 µm film thickness DB-FFAP fused silica capillary column (J&W Scientific, Folsom, CA, USA), connected to an Agilent 5973 quadrupole mass selective detector. Splitless injections were used (3 min). Helium carrier gas had a flow rate of 1.7 mL min^−1^ and the column head pressure was 12 psi. The oven was programmed to start at 50°C (1 min) and raised until 220°C (1 min) at 5°C min^−1^. The mass spectrometer was operated in the electron impact mode (EI) at 70 eV scanning the range 33–300 *m/z* at 3 scans s^−1^, in a full scan acquisition mode. The GC-qMS analysis was only applied to the five chemical standards, and their identification in all assays were confirmed by their retention times and mass spectra, which were also compared with the library data system of the GC–qMS equipment (Wiley 275).

### GC×GC–ToFMS Analysis

The SPME coating fibre containing the headspace volatile compounds of the cereals and coffee samples was manually introduced into the GC×GC–ToFMS injection port and maintained at 250°C for desorption. The injection port was lined with a 0.75 mm I.D. splitless glass liner. Splitless injections were used (30 s). LECO Pegasus 4D (LECO, St. Joseph, MI, USA) GC×GC–ToFMS system consisted of an Agilent GC 7890A gas chromatograph, with a dual stage jet cryogenic modulator (licensed from Zoex) and a secondary oven. The detector was a high-speed ToF mass spectrometer. An HP-5 30 m×0.32 mm I.D., 0.25 µm film thickness (J&W Scientific Inc., Folsom, CA, USA) was used as ^1^D column and a DB-FFAP 0.79 m x 0.25 mm I.D., 0.25 µm film thickness (J&W Scientific Inc., Folsom, CA, USA) was used as the ^2^D column. The carrier gas was helium at a constant flow rate of 2.50 mL min^−1^. The primary oven temperature was programmed from 40°C (1 min) to 140°C at 10°C min^−1^, then, from 140°C to 200°C (1 min) at 7°C min^−1^. The secondary oven temperature program was 15°C offset above the primary oven. The MS transfer line temperature was 250°C and the MS source temperature was 250°C. The modulation time was 5 s; the modulator temperature was kept at 20°C offset (above primary oven). Also, the hot and cold pulse duration time was 0.80 and 1.70 s, respectively. The ToFMS was operated at a spectrum storage rate of 100 spectra s^−1^. The mass spectrometer was operated in the EI mode at 70 eV using a range of *m/z* 33–350 and the detector voltage was −1695 V. Total ion chromatograms (TIC) were processed using the automated data processing software ChromaTOF (LECO) at S/N threshold 6. Contour plots were used to evaluate the general separation quality and for manual peak identification. A signal-to-noise threshold of 100 was used. In order to tentatively identify the different compounds, the mass spectrum of each compound detected was compared to those in mass spectral libraries which included an in-house library of standards, and two commercial databases (Wiley 275 and US National Institute of Science and Technology (NIST) V. 2.0 - Mainlib and Replib). Furthermore, a manual inspection of the mass spectra was done, combined with the use of additional data, such as the retention index (RI) value, which was determined according to the Van den Dool and Kratz RI equation [23]. For the determination of the RI, a C_6_– C_20_ *n*-alkanes series was used, and these values were compared with values reported in the literature for chromatographic columns similar to that used as the ^1^D column in the present work [24]–[40]. A mass spectral match factor, the tentatively identified compounds showed similarity matches >900, was set to decide whether a peak was correctly identified or not. The DTIC (Deconvoluted Total Ion Current) GC×GC area data were used as an approach to estimate the relative content of each volatile component. Reproducibility was expressed as relative standard deviation (RSD).

### Enumeration of Total Aerobic Microorganisms, Yeasts and Moulds

For each sample, three independent assays were performed, each one with three replicates. In each independent assay, three 10 g sub-samples of grain were suspended in 90 mL of Peptone Water (Merck, Darmstad, Germany). The enumeration of total aerobic microorganisms was based in the ISO standard 4833∶2003 [41]. After the preparation of the initial suspension and serial dilutions, 1 mL of each sample was pour-plated (three replicates) in Plate Count Agar (Merck4Food, Merck, Darmstad, Germany). Culture plates were incubated for 72±3 hours at 30±1 C. Following incubation, colonies were counted in the most suitable dilution and the result was calculated from the average colony counts in the three replicates and expressed as colony forming units *per* gram (CFU.g^−1^). The enumeration of yeasts and moulds was performed according to the Portuguese Standard NP 3277∶1987 [42]. Three replicates of each sample were spread-plated (0.5 and 0.1 mL aliquots) in Rose-Bengal Chloramphenicol Agar (Merck, Darmstad, Germany). Culture plates were incubated for 120±2 hours at 25±1 C. Following incubation, colonies of yeasts and moulds were counted independently in the most suitable volume. The results were calculated from the average colony counts in the three replicates and expressed as colony forming units *per* gram (CFU.g^−1^).

### Capture and Counting of Mite

Cereals grains and coffee beans were processed separately using a Berlese funnel for 24 hours in order to isolate mites in 250 mL erlenmeyers containing a alcoholic solution of 1/1 ethanol/distillate water (v/v) [43]. For each independent assay three sub-samples of 20 g each were analysed. The solution was filtered through polycarbonate membranes 1.2 µm pore size (Millipore, Bedford, USA) in a vacuum filtration manifold (Millipore, Bedford, USA). Mite counting was conducted under optical microscope (Leica DMLS, Leica Microsystems GmbH, Wetzlar, Germany). During counting, the distinction between adult (male and female), larval and nymphal stages was based on physiognomic characteristics [44]. Essentially, larva and adult mites were identified by the number of legs, where the former is six-legged and the latter is eight-legged. Moreover, the gender discrimination is based on the posterior part, where males have a concave shape, and females an irregular shape.

### Principal Component Analysis

In order to assess a possible relationship between the volatile metabolites and sample microbial contamination, PCA was applied to the auto-scaled areas of the 54 volatiles identified by HS-SPME/GC×GC–ToFMS presented in the 7 types of matrices under study (grains of rough, brown and white rice, oat, wheat, and green and roasted coffee beans), each one corresponding to three independent assays, and also to the values of microbial contamination (colonies of total aerobic microorganisms - TAM, yeasts, and moulds) [45]. The goal of this approach was to extract the main sources of variability and hence to help on the characterisation of the dataset.

## Results and Discussion

### Full-factorial Design for Optimization of SPME Experimental Parameters

**Figure 1:**
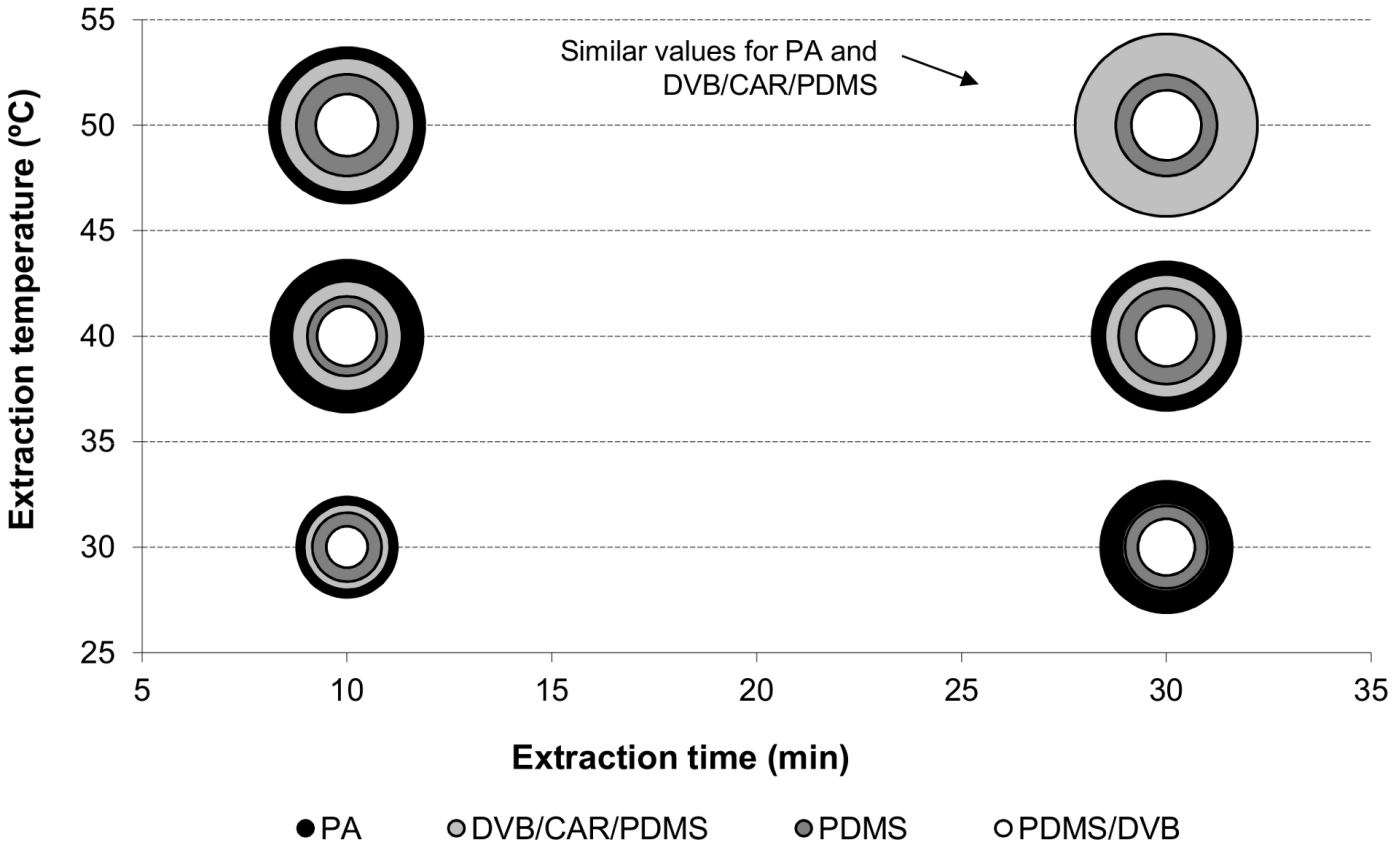
Effect of extraction temperature and time, and SPME coating fibre on the extraction efficiency. Extraction efficiency evaluated on five standards: 3-methyl-1-butanol, 3-octanone, 1-octanol, geosmin, and 1-octen-3-ol. Each bubble represents total GC peak area of the five standards.

In order to optimize the SPME procedure, a full-factorial design was implemented, which comprised the evaluation of three extraction temperatures (30.0, 40.0 and 50.0 C), two extraction times (10 and 30 min) and four coating SPME fibres (PA, PDMS, PDMS/DVB, and DVB/CAR/PDMS). The results of these analyses are represented in Fig. 1, where each bubble corresponds to the total chromatographic area of the five standards under study inherent to three different variables (extraction temperature, extraction time and the SPME fibre type). The bubble plot showed in Fig. 1 allows a straightforward comparison of the overall extraction efficiency, as a larger bubble represents a higher total chromatographic area. Independently of the used fibre, under the ranges of time and temperature studied, the higher extraction temperature and time led to higher chromatographic area, *i*.*e.* higher extraction efficiency. However, for the conditions tested, the higher extraction temperatures promoted higher GC peak areas than the higher extraction times, which suggest that the effect of the extraction temperature was more important, on the extraction efficiency, than extraction time (Fig. 1).

With the exception observed at 50.0 C, for 30 min, where PA and DVB/CAR/PDMS fibres exhibited similar chromatographic areas (Fig. 1), PA fibre generally presented the higher extraction efficiency compared to all other fibres under study. The extraction at 50.0 C, for 30 min with the SPME coating fibre DVB/CAR/PDMS was selected for further volatile metabolite microbial extractions. For these conditions, RSD was considered acceptable (9.5%). DVB/CAR/PDMS fibre was selected instead of PA fibre, because PA stationary phase retains the volatile compounds through absorption, while DVB/CAR/PDMS stationary phase has a synergistic effect between adsorption and absorption. Despite of the similar results for the two fibres, the mutually synergetic effect of adsorption and absorption of the stationary phase of the DVB/CAR/PDMS fibre creates a higher potential of retention capacity and, consequently, higher sensitivity for complex matrices, than fibres based on absorption only, namely the PA fibre. Therefore, according to the manufacturer guidance, this fibre is only recommended for polar compounds, while DVB/CAR/PDMS presents a wide range capacity of sorbing compounds with different physicochemical properties within a molecular weight ranging from 40 to 275.

### Approach for Assessment of Microbial Volatile Metabolites

In order to obtain detailed information about potential microbial volatiles, different types of cereal grains and coffee beans were analyzed by GC×GC–ToFMS, after the preliminary step of optimization of SPME experimental parameters: grains of rough, brown and, white rice, unprocessed raw oat, unprocessed raw wheat and green and roasted coffee beans. These products are not always transported and stored under the most adequate conditions which may promote the development of microbial contaminants.

**Figure 2:**
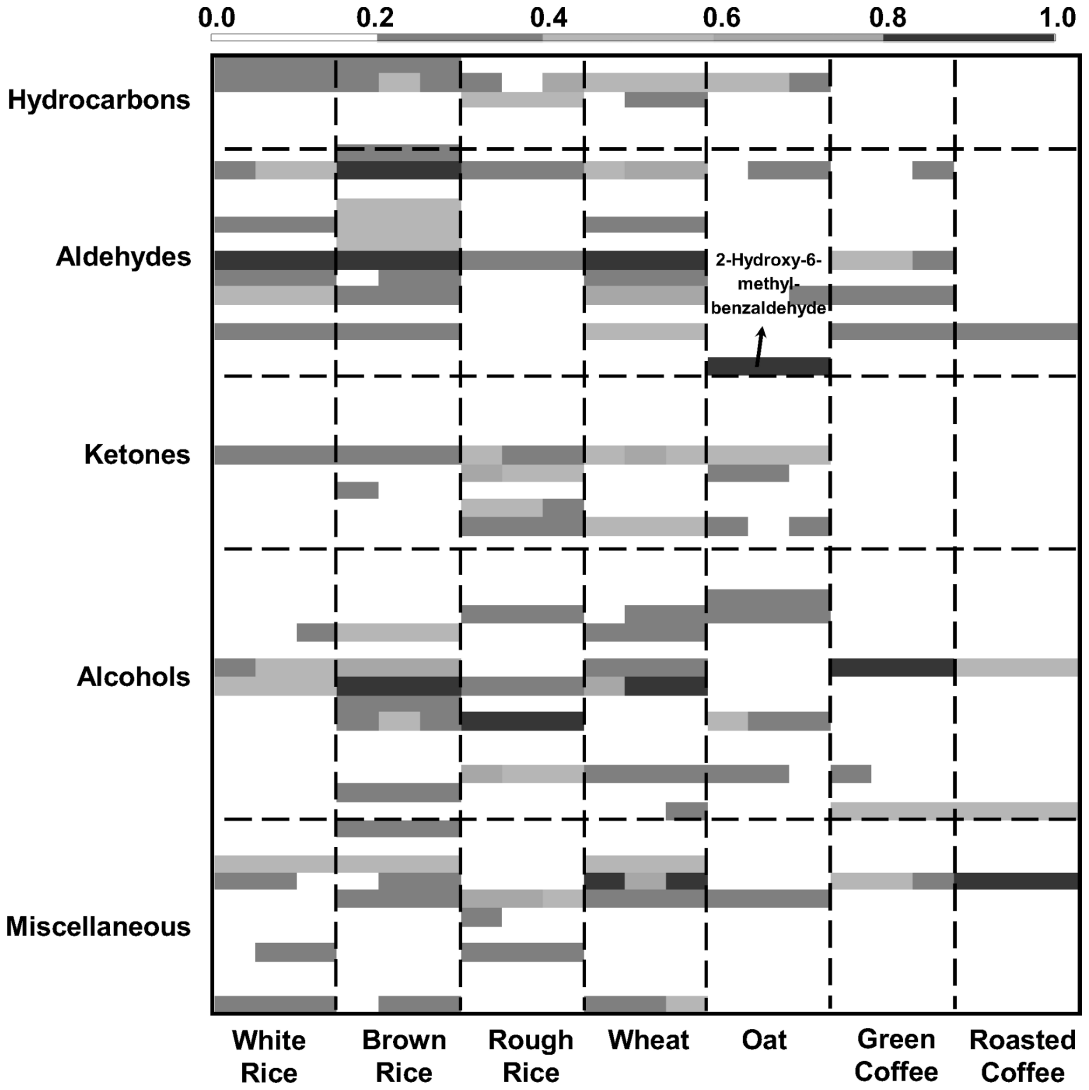
Heatmap from five cereals grains, and green and roasted coffee of 54 potential microbial markers. Different intensities correspond to the normalized GC peak areas of each compound.

From the several hundred detected compounds, only a set of 54 compounds were tentatively identified in the matrices under study (available on the supplementary data - Table S1). This set of 54 compounds was selected because 46 of them were previously reported in the literature as potential markers of microbial contamination [3], [8], [9], [12], [46]–[51], whereas the other 8 compounds (peak numbers of Table S1∶2, 5, 15, 18, 27, 34, 40, 46) have a chemical structure that may be related to microbial metabolism. Thus, the following type of compounds were also considered: *i)* short chain (≤ C_10_) alcohols, aldehydes and ketones, resultant from enzymatic breakdown of lipids and subsequent oxidations [52], *ii)* 2-enals, linked to food spoilage or degradation [48], and *iii)* terpenes, reported as taxonomic fungi markers or indicative of mycotoxin formation [52], [53]. Moreover, a heatmap was performed and illustrated on Fig. 2, for straight trough and rapid interpretation of the relative abundance of each chemical family (maximum normalization of the GC peak area) from the different analyzed samples (with three independent assays). Where for example, as rough and brown coffee present abundant potential microbial markers (>0.2 of relative abundance), it should be expected that these samples will present higher microbial load, as it can be seen on Table 1 and it will be discussed further.

**Table 1:** Total aerobic microorganisms, yeasts and moulds in three types of rice, unprocessed oat, unprocessed wheat and green and roasted coffee.

	Whiterice	RSD(%)	Brownrice	RSD(%)	Rough rice	RSD(%)	Wheat	RSD(%)	Oat	RSD(%)	Green coffee	RSD(%)	Roasted coffee	RSD(%)
Total aerobic microorganisms	1.0	0.0	383.3	5.3	1780.0	4.8	448.3	0.7	50.0	0.0	158.3	4.8	0.0	–
Moulds	11.7	4.9	1036.7	21.3	1246.7	4.9	85.0	8.4	20.0	0.0	50.0	20.0	0.0	–
Yeasts	0.0	–	0.0	–	335.7	10.0	0.0	–	1.7	15.0	0.0	–	0.0	–

Results expressed in CFU g^−1^, Mean of three independent assays, each one with three replicates (n = 9).

**Figure 3:**
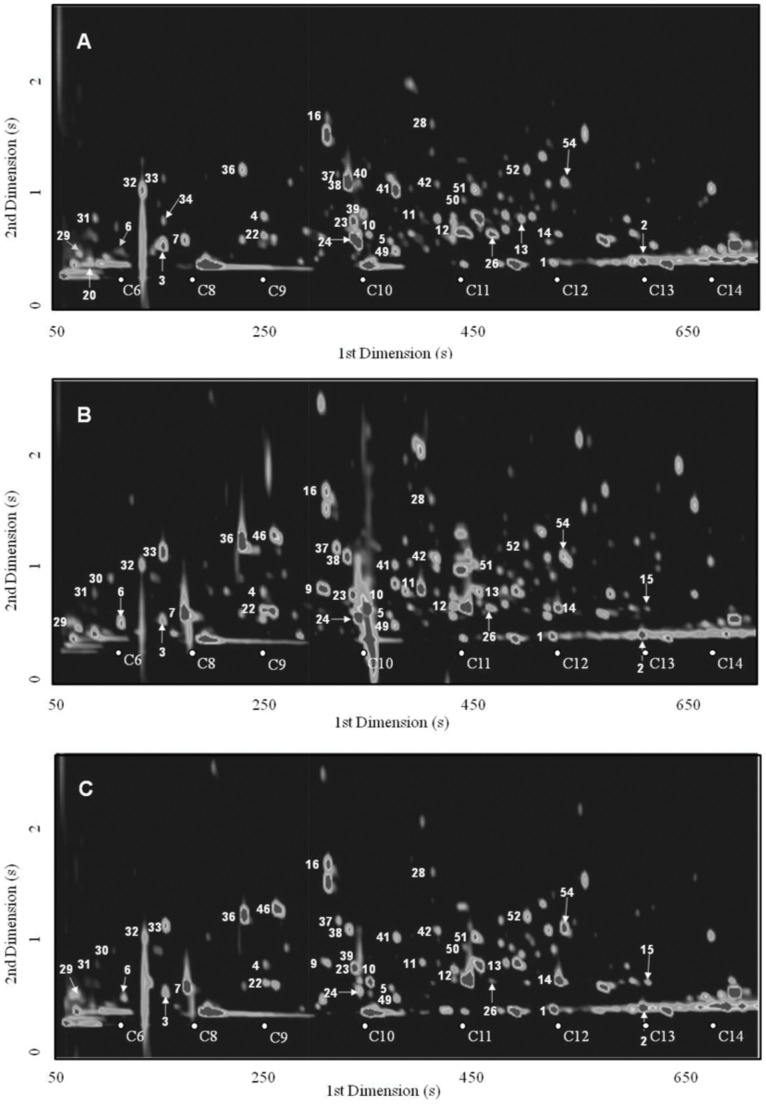
Typical GC×GC–ToFMS total ion chromatogram contour plots. Rough rice (A), brown rice (B), and white rice (C). Part of the *n*-alkanes series (C_6_–C_14_) was superimposed on the contour plots. Compounds are numbered according to Table S1.

The most reliable way to confirm the identification of each compound is based on authentic standard co-injection, which in several cases is economically prohibitive, and often unachievable in the time available for analysis, or because standards are not commercially available. Full data matrix is provided as Supplementary Data (Table S1), which include a list of the 54 selected metabolites, and the corresponding retention times in both dimensions, the retention index (RI) obtained through the modulated chromatogram and the RI reported in the literature for one dimensional GC with a 5%-Phenyl-methylpolysiloxane GC column or equivalent and for a comprehensive GC×GC system with HP-5 for the first dimension. These chromatographic data is crucial for identification purposes. Furthermore, GC×GC is an ideal technique for the analysis of complex mixtures where compounds of similar chemical structure are grouped into distinct patterns in the 2D chromatographic plane providing useful information on both their boiling point and polarity (if NP/P set of columns is used), and relationships of structured retentions have proved especially useful for compound identification (Fig. 3). This unique peculiarity of the GC×GC Chromatograms is a powerful tool in the identification step.

For example, Fig. 3 (A–C) shows the total ion chromatogram contour plots obtained from rough rice (Fig. 3A), brown rice (Fig. 3B) and white rice (Fig. 3C). This figure is displayed as an example of structured 2D contour plots observed as a result of differences in volatility inherent to the ^1^D, and the polarity on the ^2^D. Through Table S1 and Fig. 3, it is possible to identify the different chemical groups, *i.e.*, aliphatic hydrocarbons have the lower polarity, therefore have the lower retention time on the ^2^D (^2^ *t*_R_ 0.440–0.450 s), followed by aromatic hydrocarbons (^2^ *t*_R_ 0.560–0.620 s), and the increasing number of carbons on the carbon chain increases the ^1^ *t*_R._ The differences in polarity are also observable for aldehyde, ketone and alcohol groups, because they have higher ^2^ *t*_R_ (0.530–1.80 s, 0.450–1630 s, and 0.560–3.330 s, respectively), *i.e.*, higher polarity than hydrocarbons. Within these chemical families, the aromatic components presented higher ^2^ *t*_R_ than the aliphatic ones, which is in accordance with the results previously reported in literature [20]. Based on the functional groups of the chemical families under study, the ^2^ *t*_R_ values increased in this order, alkyl<aryl<aldehydes ≈ ketones ≤ alcohols, as it was previously observed [54].

The 54 compounds were tentatively identified by comparison of their mass spectra to reference database (MS) and chemical standards, when available, and by comparison of the RIs calculated (RI_calc_) with the values reported in the literature (RI_lit_) for 5% phenylpolysilphenylene-siloxane (or equivalent) column (Table S1). A range between 0 and 26 (|RI_calc_-RI_lit_|) was obtained for RI_cal_ compared to the RI_lit_ reported in the literature for one dimensional GC with 5%-phenyl-methylpolysiloxane GC column or equivalent. This difference in RIs (|RI_calc_-RI_lit_|) is considered reasonable (<4%) if one takes into account that RI_lit_ values were determined in a ^1^D chromatographic separation system, and the modulation causes some inaccuracy in ^1^D retention time (comparison with RI_calc_). In addition, comparative literature data are obtained from a large range of GC stationary phases (several commercial GC columns are composed of 5% phenyl polysilphenylene-siloxane or equivalent stationary phases) [19], which have a slight different separation selectivity than DB-FFAP, *i.e.*, the second column of the GC×GC system.

Aldehydes presented the higher chromatographic areas in white rice, brown rice and wheat, alcohols prevailed in rough rice and green coffee, aldehydes/alcohols predominate in oat grains and alcohols/miscellaneous (namely, butyrolactone) in roasted coffee, as can easily be seen on Fig. 2, and with more detail on Table S1. This is in accordance with the literature [12]. The reproducibility, expressed in RSD, of the different identified volatile compounds ranged from 0.2% to 55.8%, which is common for natural products, since they exhibit a great intrinsic variability in composition, and possibly due to the heterogeneity of the microbial spoilage, even in the same lot. The highest variability was usually observed for the trace components.

From the five tested standards (3-octanone, 1-octanol, 1-octen-3-ol, 3-methyl-1-butanol, and geosmin) only geosmin was not detected in the cereal grains and coffee beans under study. Otherwise, the other standards were identified in all food matrices. Geosmin is produced by soil *Actimomycetes* [55] and is related to unpleasant earthy/musty notes in various types of foods [49]. Furthermore, from the most common volatile metabolite microbial potential markers referred in literature (*c.f.* introduction*)*, 2-methyl-1-propanol, 3-methyl-1-butanol, 1-octanol, 1-octen-3-ol, 2-butanone, 3-octanone, 2-hexanone, 2-heptanone, limonene, dimethyl disulfide, 2-methylisoborneol, geosmin, and 3-methylfuran, only the last three were not detected in any of the matrices under study.

### Volatile Microbial Metabolites as Potential Markers for Microbial Contamination

The results of the enumeration of yeasts, moulds and total aerobic microorganisms presented in Table 1 showed a wide range of contamination levels in the different food matrices under study. Rough rice showed the highest degree of contamination, and the roasted coffee, the lowest one, without any detectable microbial contaminants. In fact, for the case of coffee beans, the most commonly reported health problems associated with the intake of roasted coffee products are not directly related to the spoilage itself, but rather associated to contamination of the raw material, green coffee beans (according Table 1, green coffee beans exhibited some degree of microbial contamination), from which hazardous thermoresistant mycotoxins may result, commonly ochratoxins, aflatoxins, sterigmatocystin and/or patulin [56], [57]. These toxins can remain unaltered or only slightly altered (but sill with toxic activity) after the thermal treating [58]. These toxins usually present health concerns, namely potential carcinogenic, immunosuppressive, teratogenic and mutagenic activities [59]. As a systematic preventive approach, a rapid screening method for the evaluation of the contamination level of the green coffee beans raw material during storage and/or transportation is imperative.

**Figure 4:**
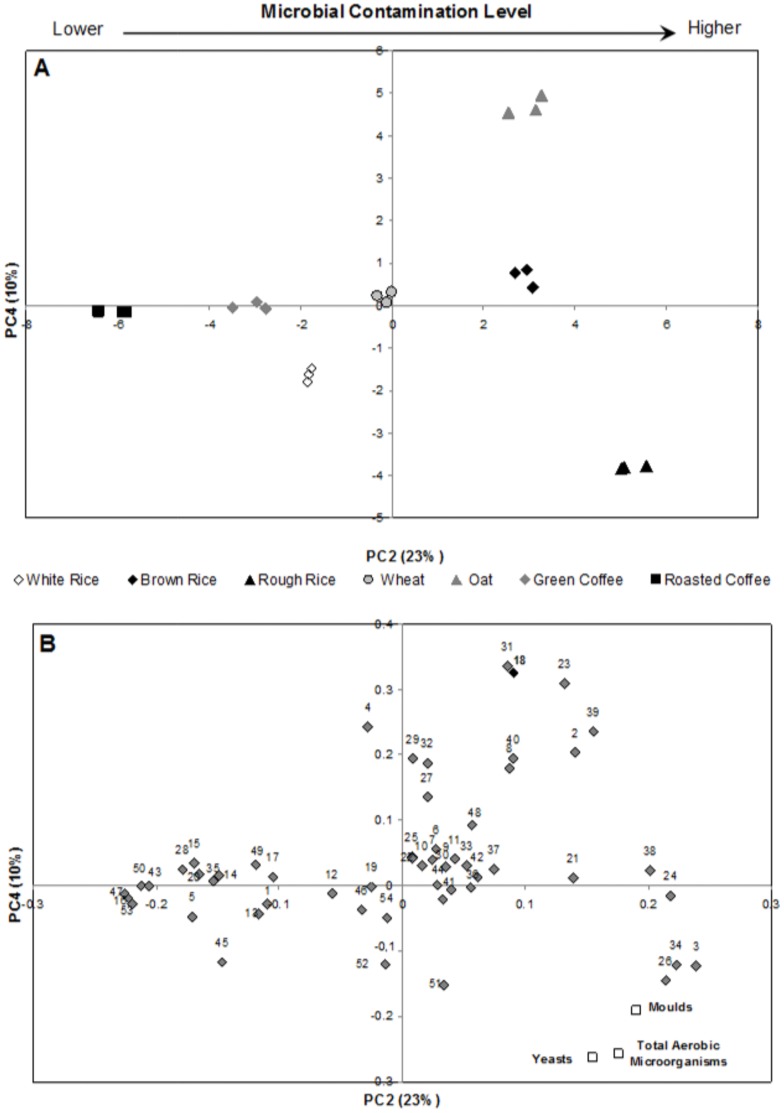
PC2×PC4 scores (A) and loadings (B) scatter plots. Compounds are numbered according to Table S1.

The approach used in the present work was to perform PCA on the merged data from the potential volatile markers of each matrix and from microbial contaminants (TAM, yeasts and moulds). Fig. 4A shows the scores scatter plot of the second (PC2) and the fourth component (PC4), which contains 33% of the total variability of the data set. A high variability, which is commonly found using the first’s principal components, as PC1 versus PC2 or PC3, was not expected because that type of variability is mainly driven by the natural volatile profile of the studied samples and not from the microbial spoilage itself.

The samples are distributed along the PC2 axis, according to their overall degree of contamination (Table 1 - total aerobic microorganisms (TAM), moulds and yeasts), from the less contaminated sample, roasted coffee, corresponding to negative scores on PC2, to the most contaminated sample, rough rice, corresponding to positive scores on PC2 positive. Unprocessed oat grains were an exception. This sample was not located on PC2 accordingly to its microbial contamination level, but rather on the quadrant corresponding to positive scores for both, PC2 and PC4. This result was related to the high abundance of 6-methyl-5-hepten-2-one (peak number 23), 2-methyl-1-propanol (peak number 31), and especially 2-hydroxy-6-methylbenzaldehyde (peak number 18) in the oat sample compared to other matrices, which positioned the unprocessed oat at positive PC2/PC4. From the three detected compounds in this sample, 2-hydroxy-6-methylbenzaldehyde is intrinsically linked to mite contamination (see section below, *2-Hydroxy-6-methylbenzaldehyde for Mite Detection* for further discussion). Fig. 4B presented the results of the respective PCA loading plots, where the peaks number 3, 21, 24, 26, 34, 38 correspond to methylbenzene, 2-hexanone, 3-octanone, 2-nonanone, 2-methyl-3-pentanol, 1-octen-3-ol, respectively. These compounds characterise the samples with highest microbial contamination level, especially rough rice.

**Figure 5:**
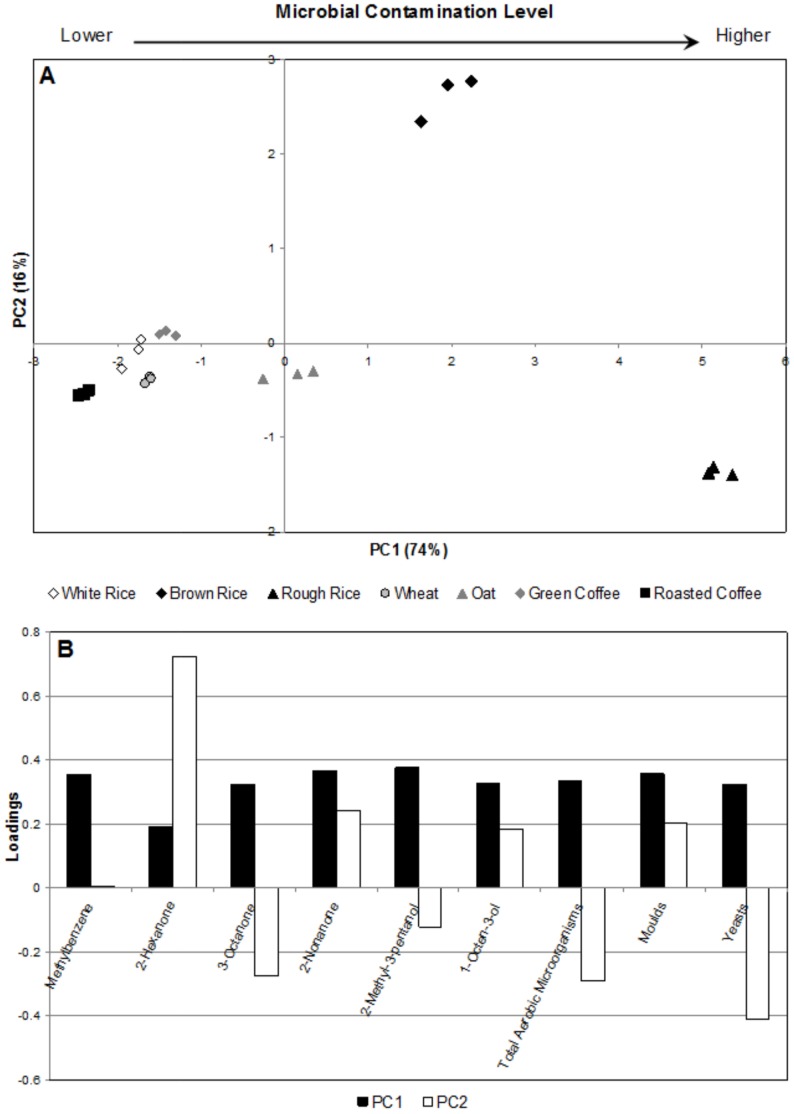
PC1×PC2 scores scatter plot (A) and loadings profiles (B) plots of six selected compounds. The selected compounds are related to the higher contamination levels: methylbenzene, 2-hexanone, 3-octanone, 2-nonanone, 2-methyl-3-pentanol, and 1-octen-3-ol.

A second PCA was performed only with these six compounds for the fully distinction between higher and lower contamination level. Fig. 5A represents the scores scatter plot of the first (PC1) and the second component (PC2), which contains 90% of the total variability. PC1 axis seems related to the microbial contamination level: samples with lower contamination (roasted coffee, white rice, green coffee, wheat and oat) were located at PC1 negative, otherwise, samples with higher microbial level (brown and rough rice) were located at PC1 positive. It was expected a wide range of contamination levels due to the type of samples under study. For instance, thermal processed samples as roasted coffee revealed no contamination level as they suffer rough thermal treatment, while raw cereals as rough rice presented a higher contamination level. Moreover, a distinction along PC2 was observed within the samples with highest contamination levels: brown and rough rice. This distinction was based on yeasts, moulds and TAM data. Rough rice, characterized by a high concentration of TAM (1780.0 CFU g^−1^), moulds (1246.7 CFU g^−1^) and yeasts (335.7 CFU g^−1^) is positioned in negative PC2, and brown rice, characterized by a lower level of TAM (383.3 CFU g^−1^) and moulds (1036.7 CFU g^−1^) and yeasts (undetected), is located at on the positive side of PC2 (Fig. 5A). Consequently, 3-octanone and 2-methyl-3-pentanol might be associated to TAM and yeasts contamination by the fact that they had ruled the position of rough rice in the negative side of PC2 axis. On the other hand, as moulds contributed positively to PC2, as well as 1-octen-3-ol, 2-nonanone and 2-hexanone, these compounds may be related to moulds. The observed distinction, based on the GC peak area of the sub-set of six compounds, allows to infer that the discrimination between samples was associated to microbial related-metabolites rather than to the volatile profile of the cereals/coffee *per se*. These observations are consistent with literature [3], [9], as these compounds (except 2-methyl-3-pentanol) were already linked to microbial spoilage.

Finally, it may be pointed out that with the application of a PCA within the selected set of the proposed markers, it was possible to cluster the different types of matrices under study, based on the level microbial of contamination (Fig. 5). Moreover, with this selection approach, a better distinction and characterization accordingly to contamination degree was achieved.

### 2-Hydroxy-6-methylbenzaldehyde for Mite Detection

**Figure 6:**
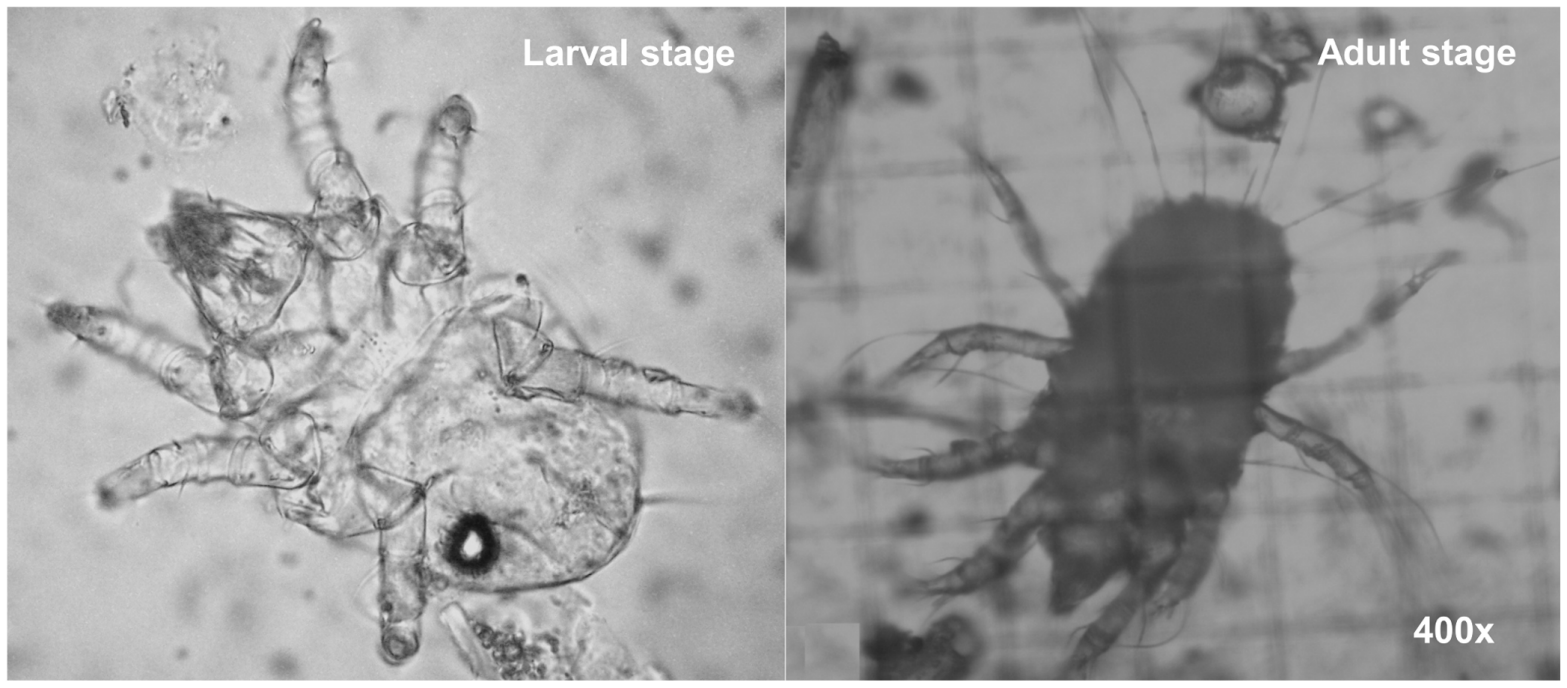
Optical micrography of mites (larval and adult stages) found in oat grains.

**Figure 7:**
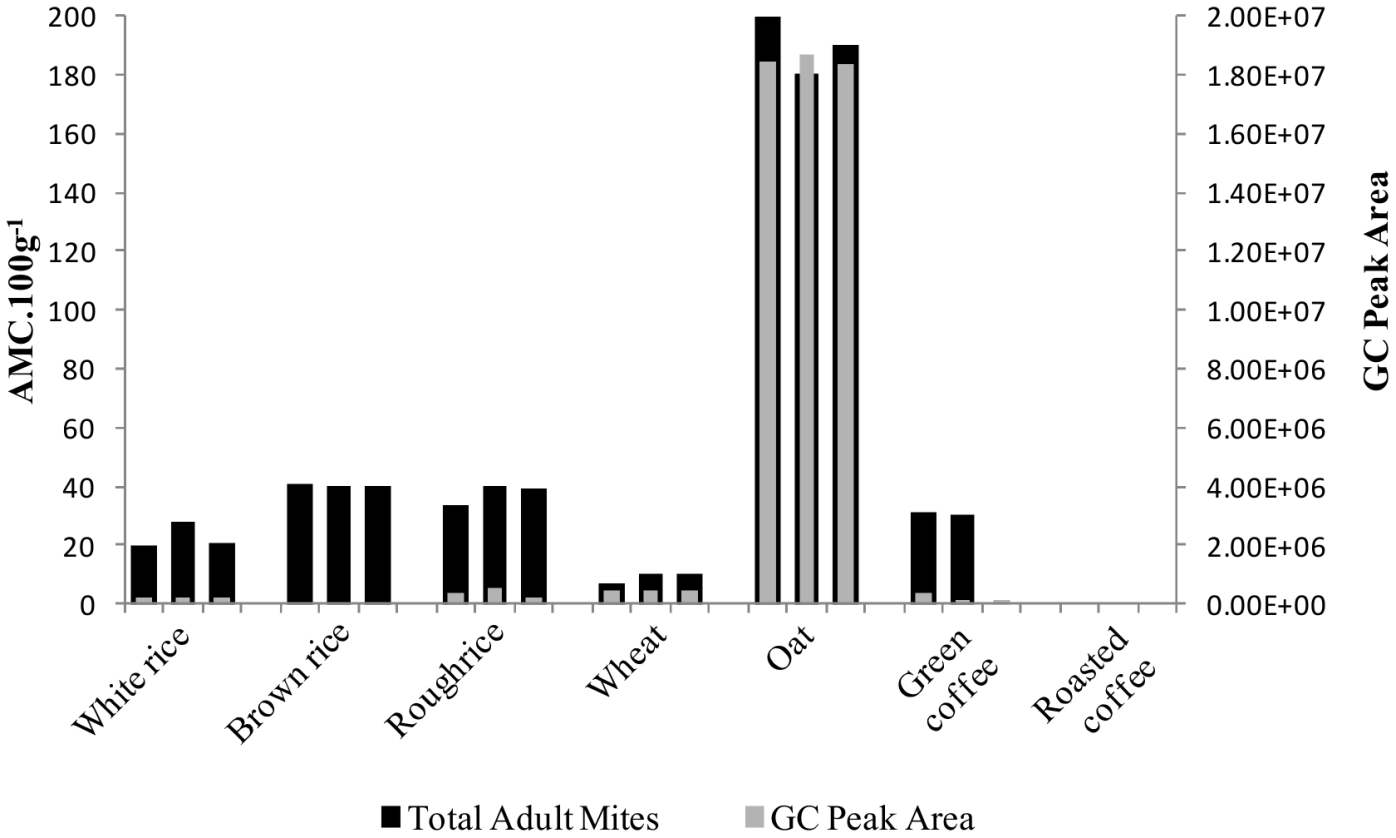
Adult mite counting *per* 100 g (AMC.100 g^−1^) and GC peak area of 2-hydroxy-6-methylbenzaldehyde.

2-Hydroxy-6-methylbenzaldehyde was a trace compound for all matrices under study, with the exception of the oat grains where a GC peak area was 1.85×10^7^ (Table S1, peak number 18 and pointed in Fig. 2). This compound has been reported as biological intermediate from several adult mite species that plays a role in alarm pheromone and sexual behaviour mediator [60], [61]. In order to relate the GC peak area of 2-hydroxy-6-methylbenzaldehyde with the presence of mites, mite counting was performed in all samples using an optical microscope (Fig. 6), and the results are presented on Fig. 7, in which presents the relation between the adult mite counting (AMC) and the GC peak area of 2-hydroxy-6-methylbenzaldehyde for the samples under study.

Microscopically, the morphological characteristics of the life stages corresponded to the larvae, and male/female adults can be distinguished (Fig. 6). As this pheromone is produced only in adult stage [61], only adult specimens were considered for the correlation of the GC peak area of the concerned compound, that represent *ca*. 70% of all mite population detected. Fig. 7 revealed that the samples under study varied in a range of 0 to 190 adult mites *per* 100 g of sample (AMC 100 g^−1^). Oat grains exhibited the highest content of adult mites (190 AMC 100 g^−1^), which was related to the highest GC peak area of 2-hydroxy-6-methylbenzaldehyde, while for the other matrices the low content of adult mite *per* 100 g (0–40) led to a lower GC peak area.

The values obtained for adult mite counting (Fig. 7) are within the values reported in literature for cereal-based foods, wherein a wide range of mites can be present [62]–[64]. For example, after domestic storage of cereal-based foods, 62% of the samples did not shown mite contamination, and only 5% of the samples presented more than 100 mites 100 g^−1^, where the maximum of detected mites was 1875 100 g^−1^ [63]. Also, in wheat flour consumed by humans the level of mites was fairly higher, reached the 5200 mites 100 g^−1^ [64]. These tremendous high level of mites in foods, could lead to problems related to mite allergens, that in the reported study, provoked anaphylactic reactions, which is especially expressed by patients that suffer respiratory allergies to mites [64]. U.S. Food and Drug Administration, recognizes the risk of ingestion mites in foods and states the need to focus the research on the health associated problems, by the fact, that those can easily induce allergic reactions in sensitized individuals [65]. Moreover, despite the dose/response of inhaled allergens of mites is well defined, until now, no consensus of dose/response of ingested allergenic mites has been achieved to scientifically determine what levels might provoke an allergic reaction [65]. Assuming that mite contamination is more than aesthetic problem, and considering the high level of sensitized individuals to mites in developed countries, the implementation of an easy and rapid approach for screening mite contamination level in foods is an actual and imperative challenge.

### Conclusions

HS-SPME/GC×GC–ToFMS is proposed as a potential tool for the parallel assessment of microbiological and mite contamination, directly in cereals grains and coffee beans, achieving a significant reduction in time of analysis, compared to standard microbial and mite count methods. Specifically, 70 min are required for the complete HS-SPME/GC×GC–ToFMS analysis (extraction plus GC analysis) which is substantially lower than the time requires by the conventional plate-count approach (about 72 to 120 h for bacteria and fungi, respectively), and mite isolation (*ca* 24 h). HS-SPME/GC×GC–ToFMS methodology was used to analyse five types of cereal grains as well as green and roasted coffee. As result, 54 potential microbial volatile metabolites reported in literature or compounds structurally associated to those. Due to its orthogonal properties, GC×GC–ToFMS reduced co-elution and improved the quality of the selection of volatile compounds potentially related to the microbial contamination. The application of PCA to analyse results obtained by different methodological approaches (GC areas and microbial counts) confirmed that the level of microbiological contamination can be inferred from the profile of volatile metabolites. Furthermore, the sub-set of six compounds (methylbenzene, 3-octanone, 2-nonanone, 2-methyl-3-pentanol, 1-octen-3-ol, and 2-hexanone) considered as microbial related-metabolites contributed for few advantages of the proposed approach: *i)* may increase the specificity of the methodology, as the selected sub-set is associated to microbiological contamination, rather than to the intrinsic volatile profile of the cereal grains and coffee beans; and *ii)* reduce the complexity of the analysis, allowing a rapid access of information about microbial contamination. However, the application of HS-SPME/GC×GC–ToFMS to the assessment of mite contamination is the major novelty. This new approach can be regarded as the basis for the development of a rapid and versatile, method that can be applied in industry to the simultaneous assessment the level of microbiological contamination and for detection of mites in cereals grains and coffee beans, and may be extended to other solid matrices.

## Supporting Information

Table S1 Potential microbial volatile compounds identified by GC×GC–ToFMS in grains of 3 types of rice, oat and wheat and in green and roasted coffee beans.
(DOC)
