# Supplementary material for: Can Volatile Organic Metabolites Be Used to Simultaneously Assess Microbial and Mite Contamination Level in Cereal Grains and Coffee Beans?
Source: PLoS One. 2013 Apr 16;8(4):e59338. doi: 10.1371/journal.pone.0059338 (PMC3628908; doi:10.1371/journal.pone.0059338)
Supplement: Table S1 — Potential microbial volatile compounds identified by GC×GC–ToFMS in grains of 3 types of rice, oat and wheat and in green and roasted coffee beans. (DOC) [file pone.0059338.s001.doc]

**Table S1**

Potential microbial volatile compounds identified by GC × GC–ToFMS in grains of 3 types of rice, oat and wheat and in green and roasted coffee beans

| **Peak number** | **^1^*t*_R_ ^a^**  **(s)** | **^2^*t*_R_ ^a^**  **(s)** | **Compound** | **RI_lit._^b^** | **RI_calc_^c^** | **White rice** | **Brown rice** | **Rough rice** | **Wheat** | **Oat** | **Green coffee** | **Roasted coffee** | **Ref^g^** |
| --- | --- | --- | --- | --- | --- | --- | --- | --- | --- | --- | --- | --- | --- |
|  |  |  |  |  |  | **Peak Area^d^ (x10^6^) and RSD (%)** | | | | | | |  |
|  |  |  | **Hydrocarbons** |  |  |  |  |  |  |  |  |  |  |
|  |  |  | ***Aliphatics*** |  |  |  |  |  |  |  |  |  |  |
| 1 | 525 | 0.440 | Dodecane | 1200 | 1201 | 4.57 (15.9) | 5.00 (3.8) | 1.06 (10.6) | 1.50 (38.9) | 1.39 (22.2) | 3.18 (23.5) | 3.65 (23.3) | [8] |
| 2 | 610 | 0.450 | Tridecane | 1299 | 1301 | 5.03 (26.6) | 8.05 (11.9) | 3.71 (82.7) | 5.03 (8.9) | 7.66 (14.9) | 5.05 (19.7) | 1.39 (33.1) | - |
|  |  |  | ***Aromatics*** |  |  |  |  |  |  |  |  |  |  |
| 3 | 155 | 0.560 | Methyl-benzene | 770 | 776 | 2.07 (9.3) | 3.36 (3.2) | 4.88 (7.5) | 2.34 (5.7) | 2.77 (18.9) | 2.51 (17.5) | 0.64 (12.2) | [9] |
| 4 | 250 | 0.840 | Styrene | 891 | 895 | 0.99 (20.2) | 1.31 (6.7) | 1.28 (8.3) | 0.71 (0.9) | 3.07 (7.4) | 1.11 (11.5) | 2.74 (14.2) | [9] |
| 5 | 370 | 0.620 | 1-Methyl-3-(1-methylethyl)-benzene | 1023 | 1026 | 0.75 (5.8) | 1.03 (4.3) | 0.61 (8.8) | 0.48 (5.6) | 0.45 (12.1) | 0.75 (6.2) | 1.92 (14.9) | - |
|  |  |  | **Subtotal (GC Peak Area)** |  |  | 13.41 | 18.76 | 11.53 | 10.07 | 15.34 | 12.60 | 10.34 |  |
|  |  |  | **Subtotal (%)** |  |  | 9.97 | 6.91 | 14.39 | 7.52 | 13.38 | 4.89 | 5.47 |  |
|  |  |  | **Aldehydes** |  |  |  |  |  |  |  |  |  |  |
|  |  |  | ***Aliphatics*** |  |  |  |  |  |  |  |  |  |  |
| 6 | 115 | 0.530 | Pentanal | 722 | 726 | 0.72 (43.6) | 5.82 (4.3) | 0.37 (12.3) | 0.95 (17.4) | 0.59 (4.3) | 1.28 (18.0) | 1.74 (0.5) | [46] |
| 7 | 175 | 0.630 | Hexanal | 800 | 801 | 7.83 (17.5) | 21.40 (5.9) | 3.00 (10.7) | 7.01 (5.7) | 3.99 (10.0) | 9.47 (37.8) | 5.37 (39.7) | [9] |
| 8 | 215 | 0.830 | 2-Hexenal | 857 | 851 | 0.29 (19.9) | 1.55 (3.4) | - | 0.69 (9.8) | 0.62 (12.9) | 0.51 (15.7) | - | [48] |
| 9 | 310 | 0.840 | 2-Heptenal | 964 | 962 | 1.26 (12.1) | 10.72 (2.1) | 0.40 (3.9) | 0.94 (10.8) | 0.14 (13.2) | 1.01 (6.4) | 2.65 (16.1) | [48] |
| 10 | 350 | 0.670 | Octanal | 1023 | 1006 | 3.90 (4.8) | 11.50 (4.7) | 0.88 (3.0) | 3.17 (2.1) | 1.28 (10.6) | 6.83 (6.5) | 1.25 (17.8) | [9] |
| 11 | 400 | 0.840 | 2-Octenal | 1056 | 1056 | 0.99 (5.5) | 11.26 (10.7) | 0.34 (4.4) | 1.10 (3.4) | 0.31 (3.8) | 1.68 (3.3) | 2.21 (16.2) | [48] |
| 12 | 445 | 0.680 | Nonanal | 1104 | 1101 | 16.56 (6.6) | 22.64 (3.4) | 2.35 (12.8) | 11.65 (4.5) | 3.24 (6.0) | 21.71 (9.8) | 5.37 (8.6) | [9] |
| 13 | 490 | 0.830 | 2-Nonenal | 1162 | 1157 | 4.59 (10.4) | 4.53 (8.9) | 0.30 (12.4) | 4.32 (4.6) | 0.44 (14.5) | 3.36 (5.9) | 2.67 (18.8) | [48] |
| 14 | 530 | 0.690 | Decanal | 1204 | 1207 | 8.81 (2.8) | 7.41 (2.7) | 1.11 (45.7) | 7.26 (2.3) | 3.18 (19.1) | 11.21 (5.9) | 5.16 (22.8) | [8] |
| 15 | 615 | 0.680 | Undecanal | 1305 | 1307 | 1.07 (3.9) | 0.83 (9.6) | 0.17^e^ | 1.02 (13.5) | 0.40 (23.6) | 1.26 (10.5) | 0.72 (31.9) |  |
|  |  |  | ***Aromatics*** |  |  |  |  |  |  |  |  |  |  |
| 16 | 310 | 1.690 | Benzaldehyde | 961 | 963 | 5.36 (10.7) | 7.40 (1.9) | 0.96 (8.8) | 5.74 (3.4) | 0.98 (5.6) | 11.23 (10.3) | 11.59 (8.9) | [9] |
| 17 | 390 | 1.720 | Benzeneacetaldehyde | 1043 | 1047 | 0.49 (6.1) | 0.80 (3.4) | 0.22 (7.5) | 1.57 (1.2) | 0.39 (1.2) | 5.61 (7.7) | 0.69 (22.0) | [9] |
| 18 | 525 | 1.800 | 2-Hydroxy-6-methyl-benzaldehyde | 1199 | 1202 | 0.19 (4.2) | - | 0.39 (42.1) | 0.43 (3.9) | 18.50 (0.9) | 0.23 (55.8) | - |  |
|  |  |  | **Subtotal (GC Peak Area)** |  |  | 52.06 | 105.85 | 10.37 | 45.87 | 34.07 | 75.39 | 39.40 |  |
|  |  |  | **Subtotal (%)** |  |  | 38.72 | 38.99 | 12.95 | 34.26 | 29.73 | 29.28 | 20.85 |  |
|  |  |  | **Ketones** |  |  |  |  |  |  |  |  |  |  |
|  |  |  | ***Aliphatics*** |  |  |  |  |  |  |  |  |  |  |
| 19 | 120 | 1.650 | 3-Hydroxy-2-Butanone | 727 | 733 | 0.46 (23.8) | 1.32 (8.7) | 0.17 (20.7) | 0.15 (3.9) | 0.09 (1.4) | 1.88 (10.3) | - | [9] |
| 20 | 85 | 0.450 | 2-Butanone | 712 | 688 | - | 0.65 (6.2) | 0.30 (13.9) | 0.25 (8.6) | 0.49 (7.2) | 0.48 (28.8) | 4.14 (17.6) | [9] |
| 21 | 170 | 0.720 | 2-Hexanone | 788 | 795 | 0.07 (32.5) | 0.39 (7.3) | 0.13 (5.9) | 0.07 (11.2) | 0.08 (3.1) | 0.11 (4.78) | - | [9] |
| 22 | 250 | 0.660 | 2-Heptanone | 882 | 895 | 0.36 (9.0) | 4.11 (6.3) | 0.86 (0.9) | 0.58 (9.9) | 0.76 (2.1) | 0.67 (2.7) | 2.46 (10.5) | [9] |
| 23 | 335 | 0.800 | 6-Methyl-5-hepten-2-one | 974 | 990 | 4.42 (11.9) | 6.19 (9.6) | 3.80 (20.8) | 7.16 (10.7) | 10.02 (7.7) | 4.78 (8.1) | 2.05 (19.2) | [8] |
| 24 | 335 | 0.640 | 3-Octanone | 986 | 990 | 0.35 (15.9) | 1.31 (7.5) | 5.28 (5.0) | 0.20 (2.4) | 3.94 (8.3) | 0.41 (7.6) | - | [3] |
| 25 | 385 | 0.830 | 3-Octen-2-one | 1036 | 1041 | 0.27 (10.0) | 4.49 (7.9) | 0.17 (20.0) | 0.51 (2.3) | 0.19 (4.3) | 0.58 (8.8) | 1.80 (13.1) | [3] |
| 26 | 465 | 0.680 | 2-Nonanone | 1102 | 1126 | 0.78 (14.6) | 3.52 (5.7) | 3.98 (0.4) | 0.40 (18.2) | 1.14 (17.9) | 0.96 (7.5) | - | [9] |
| 27 | 730 | 0.850 | 5,9-Undecadien-2-one, 6,10-dimethyl | 1458 | 1451 | 1.75 (3.3) | 1.36 (26.3) | 2.39 (6.5) | 5.55 (14.7) | 3.86 (10.9) | 1.63 (13.9) | 2.62 (22.1) |  |
|  |  |  | ***Aromatics*** |  |  |  |  |  |  |  |  |  |  |
| 28 | 410 | 1.630 | 1-Phenyl-ethanone | 1065 | 1067 | 1.10 (29.9) | 1.50 (1.6) | 0.69 (3.2) | 0.95 (11.0) | 1.40 (5.6) | 1.08 (21.5) | 7.40 (13.2) | [12] |
|  |  |  | **Subtotal (GC Peak Area)** |  |  | 9.55 | 24.84 | 17.77 | 15.83 | 21.97 | 12.60 | 20.47 |  |
|  |  |  | **Subtotal (%)** |  |  | 7.10 | 9.15 | 22.18 | 11.82 | 19.17 | 4.89 | 10.83 |  |
|  |  |  | **Alcohols** |  |  |  |  |  |  |  |  |  |  |
|  |  |  | ***Aliphatics*** |  |  |  |  |  |  |  |  |  |  |
| 29 | 70 | 0.560 | Ethanol | 668 | 669 | 1.46 (34.9) | 2.19 (2.4) | 0.30 (20.2) | 1.16 (12.2) | 1.37 (16.7) | 0.94 (5.1) | 0.96 (4.9) | [9] |
| 30 | 105 | 0.950 | 1-Butanol | 688 | 714 | 0.54 (30.6) | 1.67 (2.5) | 0.14^e^ | 0.50 (7.3) | 0.08^f^ (12.9) | 0.55 (8.3) | 0.13 (5.1) | [9] |
| 31 | 90 | 0.810 | 2-Methyl-1-propanol | 695 | 695 | 0.22 (38.3) | 0.92 (17.5) | 0.82 (4.5) | 0.64 (4.1) | 4.50 (4.9) | 1.24 (1.1) | 0.83 (13.6) | [3] |
| 32 | 135 | 1.050 | 3-Methyl-1-butanol | 735 | 751 | 0.62 (9.7) | 2.71 (4.4) | 2.92 (4.1) | 2.39 (7.5) | 4.80 (3.8) | 5.94 (0.8) | 2.38 (14.7) | [3] |
| 33 | 155 | 1.160 | 1-Pentanol | 766 | 776 | 2.59 (33.3) | 11.96 (1.9) | 0.58 (5.5) | 3.87 (5.6) | 0.53 (1.8) | 2.34 (8.5) | 1.32 (13.2) | [9] |
| 34 | 155 | 0.800 | 2-Methyl-3-pentanol | 772 | 776 | - | 0.30 (5.6) | 0.74 (6.7) | - | 0.28 (3.4) | - | - |  |
| 35 | 165 | 4.790 | 2,3-Butanediol | 806 | 793 | 7.79 (19.3) | 15.07 (6.2) | 0.60 (19.2) | 3.10 (16.9) | 2.37 (15.9) | 48.51 (6.0) | 19.97 (19.6) | [8] |
| 36 | 230 | 1.240 | 1-Hexanol | 865 | 870 | 8.05 (10.3) | 21.45 (6.5) | 2.63 (3.2) | 9.90 (15.6) | 1.34 (5.8) | 6.08 (0.9) | 1.61 (29.2) | [9] |
| 37 | 320 | 1.200 | 1-Heptanol | 962 | 974 | 1.14 (6.7) | 6.41 (4.2) | 0.51 (5.5) | 1.65 (1.7) | 0.27 (2.7) | 0.75 (1.6) | 0.48 (13.2) | [9] |
| 38 | 330 | 1.120 | 1-Octen-3-ol | 991 | 985 | 2.19 (14.0) | 8.82 (3.3) | 9.20 (12.1) | 1.71 (6.8) | 7.62 (10.9) | 2.68 (3.4) | 4.01 (11.3) | [3] |
| 39 | 345 | 0.870 | 3-Octanol | 993 | 1001 | 1.32 (4.2) | 0.30 (22.5) | 0.50 (20.9) | 0.17 (3.2) | 1.10 (10.4) | 0.45 (8.9) | - | [9] |
| 40 | 340 | 1.120 | 6-Methyl-5-hepten-2-ol | 1003 | 996 | 0.31 (9.5) | 0.55 (6.1) | 0.53 (1.2) | 0.69 (5.7) | 1.15 (11.6) | 1.23 (11.6) | - |  |
| 41 | 375 | 1.060 | 2-Ethyl-1-hexanol | 1032 | 1031 | 2.06 (7.0) | 1.93 (6.4) | 5.23 (0.2) | 4.09 (14.1) | 4.06 (14.1) | 8.51 (19.2) | 1.06 (6.0) | [3] |
| 42 | 415 | 1.120 | 1-Octanol | 1087 | 1071 | 0.13 (7.3) | 5.32 (2.9) | 0.65 (2.7) | 1.89 (4.6) | 0.36 (4.5) | 1.13 (2.4) | 0.72 (16.9) | [9] |
|  |  |  | ***Aromatics*** |  |  |  |  |  |  |  |  |  |  |
| 43 | 450 | 3.330 | Phenylethyl Alcohol | 1116 | 1110 | 1.09 (7.9) | 1.68 (2.8) | 0.32 (12.6) | 2.29 (5.2) | 0.89 (3.3) | 25.78 (2.9) | 21.24 (7.0) | [47] |
|  |  |  | **Subtotal (GC Peak Area)** |  |  | 29.55 | 81.29 | 25.62 | 34.04 | 30.68 | 106.12 | 54.70 |  |
|  |  |  | **Subtotal (%)** |  |  | 21.98 | 29.94 | 31.98 | 25.43 | 26.77 | 41.21 | 28.94 |  |
|  |  |  | **Miscellaneous** |  |  |  |  |  |  |  |  |  |  |
| 44 | 505 | 4.200 | Octanoic acid | 1179 | 1180 | 0.61 (8.9) | 6.88 (5.7) | 0.39 (40.2) | 1.63 (9.6) | - | 2.99 (9.4) | 0.78 (11.4) | [9] |
| 45 | 140 | 0.670 | Dimethyl disulfide | 738 | 757 | 1.46 (19.2) | 0.08 (11.2) | 0.22 (24.4) | 0.08^f^ (20.4) | 0.13 (7.9) | 0.10^e^ | 1.14 (12.2) | [3] |
| 46 | 260 | 1.320 | 2-Butoxy-ethanol | 904 | 907 | 8.47 (12.9) | 10.34 (4.9) | 0.20 (12.8) | 5.04 (3.5) | 0.12 (9.8) | 7.72 (18.5) | 0.68 (28.8) |  |
| 47 | 270 | 3.260 | Butyrolactone | 915 | 920 | 3.74 (24.3) | 4.57 (1.4) | 1.25 (23.8) | 9.35 (4.4) | 1.13 (18.5) | 19.19 (6.1) | 43.07 (4.4) | [50] |
| 48 | 340 | 0.600 | 2-Pentyl-furan | 1001 | 995 | 1.57 (6.0) | 6.34 (4.9) | 5.61 (5.0) | 2.54 (3.5) | 5.96 (3.9) | 2.36 (7.0) | 7.14 (6.1) | [3] |
| 49 | 375 | 0.530 | Limonene | 1030 | 1031 | 1.52 (11.7) | 2.61 (6.1) | 1.58 (16.2) | 0.74 (4.1) | 1.83 (11.9) | 1.46 (10.9) | 4.36 (9.1) | [9] |
| 50 | 440 | 0.980 | Linalool | 1101 | 1096 | 0.48 (4.5) | 0.57 (9.9) | 0.44 (5.8) | 0.28 (5.7) | 0.54 (5.9) | 2.95 (9.5) | 3.72 (7.6) | [47] |
| 51 | 450 | 1.060 | D-Fenchyl alcohol | 1123 | 1108 | 3.66 (6.1) | 2.89 (3.3) | 2.31 (6.3) | 1.90 (2.6) | 0.91 (8.7) | 3.86 (1.2) | - |  |
| 52 | 500 | 1.220 | Endo-borneol | 1165 | 1170 | 2.42 (13.8) | 1.09 (7.7) | 1.31 (3.6) | 1.90 (4.5) | 0.70 (7.5) | 2.82 (0.4) | - | [8] |
| 53 | 510 | 0.760 | 2-Methoxy-3-Isobutyl-Pyrazine | 1186 | 1182 | 0.25 (0.4) | 0.16 (3.4) | 0.14 (4.7) | 0.05 (5.8) | - | 1.68 (18.3) | 3.15 (7.9) | [49] |
| 54 | 535 | 1.130 | Verbenone | 1204 | 1213 | 5.69 (0.2) | 5.17 (10.9) | 1.36 (2.9) | 4.55 (2.0) | 1.22 (3.2) | 5.65 (4.9) | - | [51] |
|  |  |  | **Subtotal (GC Peak Area)** |  |  | 29.87 | 40.70 | 14.81 | 28.06 | 12.54 | 50.78 | 64.04 |  |
|  |  |  | **Subtotal (%)** |  |  | 22.22 | 14.99 | 18.49 | 20.96 | 10.97 | 19.72 | 33.89 |  |
|  |  |  | **Total** |  |  | 134.44 | 271.44 | 80.10 | 133.87 | 114.60 | 257.49 | 188.95 |  |
|  |  |  | **Number of identified compounds** |  |  | 52 | 53 | 53 | 53 | 52 | 53 | 42 |  |
| ^a^ Retention times for first (^1^*t*_R_) and second (^2^*t*_R_) dimensions in seconds. | | | | | | | | | | | | | |
| ^b^ RI, Retention Index reported in the literature for HP-5 GC column or equivalents [24-40]. | | | | | | | | | | | | | |
| ^c^ RI: Retention Index obtained through the modulated chromatogram. | | | | | | | | | | | | | |
| ^d^ Mean of three independent assays (n=3). | | | | | | | | | | | | | |
| ^e^ The compound was detected in one assay. | | | | | | | | | | | | | |
| ^f^ The compound was detected in two assays. | | | | | | | | | | | | | |
| ^g^  References reported in the literature for potential markers as microbial volatiles organic compounds. | | | | | | | | | | | | | |
